# Philadelphia Chromosome-Positive B-cell Acute Lymphoblastic Leukemia: A Case Report

**DOI:** 10.7759/cureus.105705

**Published:** 2026-03-23

**Authors:** Alireza Izadian Bidgoli, Alberto Gomez Veliz, Shabnam Yazdanpanah, Gabriel Saavedra, Amanda Pina, Guillermo Rame

**Affiliations:** 1 Medical School, American University of the Caribbean School of Medicine, Cupecoy, SXM; 2 Internal Medicine, Jackson Memorial Hospital, Miami, USA; 3 Medical School, Ross University School of Medicine, Bridgetown, BRB

**Keywords:** acute lymphoblastic leukemia (all), bcr-abl1 rearrangement, dasatinib, leukemia, philadelphia chromosome-positive b-cell acute lymphoblastic leukemia (ph+ b-all), remission

## Abstract

B-cell acute lymphoblastic leukemia (B-ALL) with the Philadelphia translocation-positive genotype (Ph+) is a high-risk leukemia subtype in older adults, where comorbidity often limits the use of intensive management and complicates clinical decision-making. We present the case of a 77-year-old female with progressive fatigue, dizziness, dyspnea, and palpitations, whose laboratory evaluation revealed symptomatic anemia and circulating blast cells. Peripheral smear and flow cytometry confirmed B-ALL, and fluorescence in situ hybridization (FISH) demonstrated BCR-ABL translocation. Cardiac examination revealed preserved systolic function with valvular disease and pulmonary hypertension, influencing treatment selection. Following a multidisciplinary tumor board discussion, the patient underwent reduced-intensity induction with mini-CVD (cyclophosphamide, vincristine, and dexamethasone) combined with dasatinib and central nervous system prophylaxis. Subsequent bone marrow examination demonstrated no morphologic or immunophenotypic evidence of residual leukemia, consistent with morphologic remission. The patient experienced clinical and hematologic improvement and was discharged on continuous tyrosine kinase inhibitor therapy with close monitoring. This case highlights the diagnostic workup, multidisciplinary treatment planning, and early therapeutic response in an elderly patient with Ph+ B-ALL while comparing these findings with the current literature.

## Introduction

Philadelphia chromosome-positive B-cell acute lymphoblastic leukemia (Ph+ B-ALL) is a high-risk lymphoid malignancy and the most common genetic subgroup of ALL in adults. It is characterized by a translocation between chromosomes 9 and 22, which fuses the *BCR-ABL1* gene, encoding a constitutively activated tyrosine kinase-signaling protein [1]. The incidence of ALL follows a bimodal distribution, with the first peak in childhood and the second around 50 years of age. The incidence of Ph+ B-ALL rises significantly among older patients [2]. Historically, ALL has excellent prognostic outcomes in children, with treatment achieving up to 80% cure rates, whereas adults and older patients have a poorer prognosis, with cure rates of approximately 40-50% with traditional treatment using hyperfractionated cyclophosphamide, vincristine, doxorubicin, and dexamethasone (hyper-CVAD). These differences are thought to be linked to factors such as tolerance of cytotoxic chemotherapy and the prevalence of comorbidities in adults. However, over the past decade, advances in treatment with tyrosine kinase inhibitors (TKIs) have significantly increased survival and remission rates in adults with Ph+ B-ALL. Despite these advances, treatment in the elderly population remains a challenge, given that advanced age, frailty, and comorbidities may limit aggressive treatment with hyper-CVAD and make elderly patients poor candidates for stem cell transplantation [3]. Reduced-intensity chemotherapy with TKIs has been shown to be an effective alternative [4]; however, limited data in patients of advanced age with significant cardiovascular comorbidities demonstrate the importance of highlighting such cases.

We report the case of a 77-year-old woman with significant comorbidities diagnosed with Ph+ B-ALL who achieved morphologic remission after induction with a reduced-intensity mini-CVD (cyclophosphamide, vincristine, and dexamethasone) regimen combined with dasatinib. This case highlights the importance of individualized treatment and supports the feasibility of TKI-based low-intensity regimens for elderly patients who may not be ideal candidates for intensive chemotherapy or transplantation.

## Case presentation

Clinical presentation

A 77-year-old female with a past medical history significant for chronic deep vein thrombosis (DVT) on anticoagulation therapy as well as hypertension, dyslipidemia, and heart failure with preserved ejection fraction (HFpEF) presented with four days of progressive fatigue, dizziness, generalized weakness, shortness of breath, and palpitations. She was initially evaluated at an outside hospital, where severe anemia (hemoglobin: 5 g/dL) was identified, and she received two units of packed red blood cells. At the time of transfusion, the patient was informed that her anemia might be due to a malignancy. The patient ultimately decided to leave and seek further care elsewhere.

Physical examination and initial assessment

Initial laboratory evaluation at presentation demonstrated anemia and thrombocytopenia with circulating blasts, raising concern for acute leukemia. Despite the absence of marked leukocytosis, the presence of circulating blasts on peripheral smear was highly suggestive of an underlying hematologic malignancy. The physical examination demonstrated an Eastern Cooperative Oncology Group Performance Status (ECOG‐PS) scale of 2 [5]. She was awake, alert, oriented, and in no acute distress. Cardiovascular examination showed normal sinus rhythm without murmurs; however, further evaluation documented bilateral lower-extremity edema (+2 to +3) with signs of chronic venous insufficiency. Lungs were clear bilaterally without respiratory distress. No palpable lymphadenopathy was present in the cervical, axillary, or inguinal regions. The abdomen was soft, nontender, and nondistended. No hepatosplenomegaly was documented. The neurological examination revealed no focal deficits. Initial workup supported the diagnosis of acute leukemia in the setting of severe anemia and leukocytosis. Hematology and oncology consultation was obtained promptly.

Radiographic and specialized studies

Baseline laboratory evaluation demonstrated hematologic abnormalities consistent with acute leukemia. Hemoglobin levels ranged from 7.7 to 9.3 g/dL during the hospitalization period following transfusion. The platelet count initially measured 102 × 10⁹/L and subsequently increased to greater than 200 × 10⁹/L after initiation of therapy. The white blood cell count ranged from 3.3 to 5.7 × 10⁹/L during treatment. Laboratory testing also revealed elevated lactate dehydrogenase levels (up to 287 U/L), suggesting increased cellular turnover. Mild transaminitis was also observed, with aspartate aminotransferase levels up to 77 U/L and alanine aminotransferase levels up to 116 U/L. Renal function remained preserved throughout the hospitalization. These laboratory findings are summarized in Table 1.

**Table 1:** Laboratory findings at presentation and during hospitalization. Values at presentation represent initial laboratory findings before the initiation of therapy. Subsequent values reflect laboratory trends during hospitalization following transfusion and treatment. Reference ranges represent typical adult laboratory values and may vary slightly depending on the performing laboratory. LDH = lactate dehydrogenase; AST = aspartate aminotransferase; ALT = alanine aminotransferase

Parameter	At presentation (pre-treatment)	During hospitalization (range)	Reference range
White blood cell count (×10⁹/L)	5.7	3.3–5.7	4.0–11.0
Hemoglobin (g/dL)	7.7	8.8–9.3	12.0–16.0
Platelets (×10⁹/L)	102	210–211	150–400
LDH (U/L)	287	204–287	140–280
AST (U/L)	77	72–74	10–40
ALT (U/L)	116	100–116	7–56

**Figure 1:**
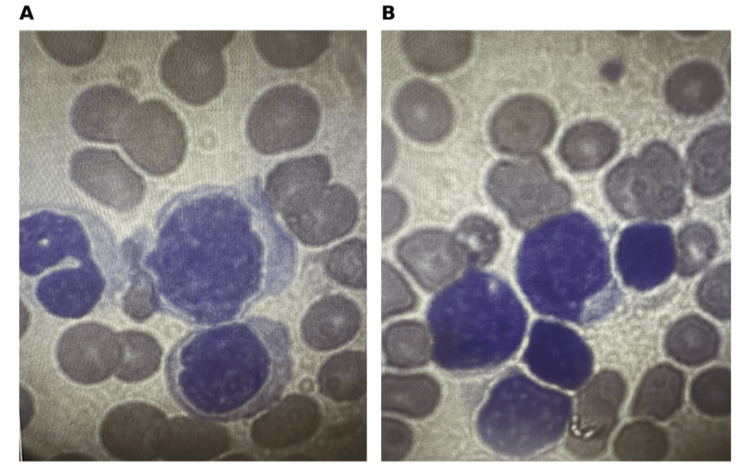
Peripheral blood smear demonstrating circulating lymphoblasts. (A, B) Representative peripheral blood smear images showing circulating blasts with high nuclear-to-cytoplasmic ratio, scant basophilic cytoplasm, and fine chromatin, consistent with acute lymphoblastic leukemia.

**Figure 2:**
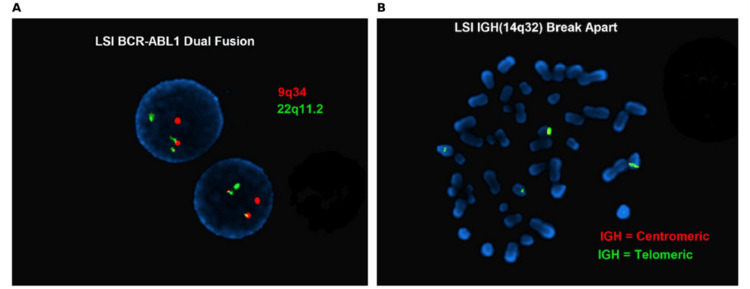
Fluorescence in situ hybridization (FISH) analysis demonstrating BCR-ABL1 fusion and IGH break-apart signals. (A) Dual-fusion FISH probe demonstrating *BCR-ABL1* rearrangement with fusion signals corresponding to the t(9;22)(q34;q11.2) translocation. Red signals represent *ABL1* (9q34) and green signals represent *BCR* (22q11.2). (B) Break-apart FISH analysis of the immunoglobulin heavy chain (IGH) locus (14q32) demonstrating separation of centromeric (red) and telomeric (green) signals, consistent with *IGH* gene rearrangement. The *IG*H locus is a common site of chromosomal translocation in B-cell malignancies, and its disruption reflects underlying genomic instability in lymphoid neoplasms. Such rearrangements may involve diverse partner genes and are frequently observed in B-cell leukemias and lymphomas, contributing to dysregulated gene expression and leukemogenesis.

Peripheral smear demonstrated circulating blasts and immature granulocytes. Peripheral blood images showed a large lymphoblast with a high nuclear-to-cytoplasmic ratio, fine chromatin, and scant cytoplasm, consistent with ALL (Figure 1). Flow cytometry of peripheral blood confirmed B-lymphoblastic leukemia or lymphoma with 54% blasts of the analyzed events. Fluorescence in situ hybridization (FISH) testing was positive for *BCR-ABL1* rearrangement, establishing Ph+ B-ALL (Figure 2).

Transthoracic echocardiography demonstrated preserved left ventricular systolic function with an estimated ejection fraction of 55-60%. Additional findings included grade I diastolic dysfunction, moderate aortic regurgitation, and mild-to-moderate tricuspid regurgitation. The estimated right ventricular systolic pressure ranged from 50 to 59 mmHg, consistent with pulmonary hypertension. Lower-extremity duplex ultrasonography revealed chronic extensive bilateral DVT with multiple segments of partial and complete venous occlusion.

First multidisciplinary tumor board discussion

The case was discussed during the tumor board review. The patient’s advanced age (77 years), ECOG-PS scale of 2, significant cardiovascular comorbidities (HFpEF, moderate valvular regurgitation, and pulmonary hypertension), and history of chronic DVT were highlighted. Cytogenetic confirmation of *BCR-ABL* positivity categorized her condition as high-risk Ph+ B-ALL. Given her frailty and comorbidities, intensive hyper-CVAD-based induction was deemed high risk. The consensus favored a lower intensity induction strategy combining mini-CVD with a TKI. Dasatinib was selected because of its central nervous system (CNS) penetration and established efficacy in Ph+ B-ALL. The goal of this therapy was induction of remission with tolerable toxicity and avoidance of excessive treatment-related morbidity. Antimicrobial prophylaxis and tumor lysis monitoring were recommended.

Tissue diagnosis

Bone marrow biopsy was attempted early during the hospitalization; the first procedure yielded a dry tap. Subsequent biopsy demonstrated hypercellular marrow (approximately 60%) with erythroid-predominant hematopoiesis following treatment initiation. No morphological or immunophenotypic evidence of residual B-ALL was identified on follow-up marrow examination. Peripheral blood showed microcytic anemia and thrombocytopenia.

The diagnostic comments stated findings consistent with remission; correlation with *BCR-ABL1* minimal residual disease testing was recommended for complete interpretation. Thus, the final pathologic diagnosis was Ph+ B-ALL, status post induction therapy with morphologic remission achieved.

Second multidisciplinary tumor board discussion

Following induction with mini-CVD plus dasatinib, the patient demonstrated hematologic recovery and clearance of morphologic disease on bone marrow biopsy. Cytarabine and methotrexate were administered for CNS prophylaxis; cerebrospinal fluid cytology and flow cytometry were negative for malignant involvement.

During the subsequent tumor board review, several key management considerations were discussed. These included confirmation of morphologic remission pending measurable residual disease testing, continuation of dasatinib-based therapy, and the development of a long-term management strategy in a septuagenarian patient who was not considered a candidate for allogeneic stem cell transplantation because of advanced age and significant comorbidities. Additional discussion focused on anticoagulation management in the setting of thrombocytopenia and chronic DVTs, as well as monitoring for potential dasatinib-associated toxicities, including QT prolongation, pleural effusion, and treatment-related cytopenias.

The multidisciplinary team reached a consensus to continue dasatinib therapy with consolidation according to a reduced-intensity protocol, maintain antimicrobial prophylaxis with acyclovir and trimethoprim-sulfamethoxazole, and perform serial *BCR-ABL1* quantitative polymerase chain reaction (PCR) testing for measurable residual disease surveillance.

Treatment response and clinical outcome

The patient tolerated induction therapy without major infectious complications. Expected cytopenias were managed with transfusion support (including red blood cell transfusions) and growth factor support during periods of neutropenia. During hospitalization, she experienced atrial fibrillation with rapid ventricular response, which was medically managed with rate control. Anticoagulation was adjusted because of thrombocytopenia, with transition planning back to a direct oral anticoagulant once platelet counts stabilized. Follow-up laboratory studies demonstrated improvement in cytopenias, with normalization of platelet counts and stabilization of hemoglobin. Bone marrow biopsy performed after induction showed no morphologic or immunophenotypic evidence of residual leukemia, consistent with remission.

At discharge, she was clinically stable, afebrile, and hemodynamically stable with improving lower-extremity edema. ECOG-PS remained 2 but was functionally improved compared with presentation. She was discharged home with home health services and close hematology follow-up. Discharge medications included dasatinib 140 mg daily along with antimicrobial prophylaxis and supportive cardiac medications.

In summary, this case describes a 77-year-old woman diagnosed with Ph+ B-ALL presenting with severe symptomatic anemia and circulating blasts on peripheral smear. Given her advanced age and comorbidities, she was successfully treated with a reduced-intensity mini-CVD plus dasatinib regimen. She achieved morphologic remission following induction therapy, highlighting the feasibility and efficacy of TKI-based, lower-intensity regimens in elderly patients with Ph+ ALL who are not candidates for intensive chemotherapy or transplantation.

## Discussion

Background (history, epidemiology, and WHO classification)

In 1986, the Philadelphia chromosomal translocation, which usually occurs in chronic myeloid leukemia (CML), was found to also occur in Ph+ ALL [6]. Although the study showed different *BCR* rearrangement patterns compared with CML, in situ hybridization and Southern blot analysis demonstrated *BCR* rearrangement and translocation of the *C-ABL* oncogene in Ph+ ALL, showing that Ph+ ALL is heterogeneous [6]. In 1987, a novel p190 ABL protein expressed in Ph+ ALL was discovered by Chan and colleagues [7]. The protein differed from the protein identified in CML, p210 [7]. In 1988, Clark and colleagues showed that the *BCR-ABL* oncogene in Ph+ ALL expressed a 7-kilobase messenger RNA encoding the P185 protein [8]. This molecular distinction established Ph+ ALL as the most common genetic subgroup of adult ALL, accounting for approximately 20-25% of cases, and historically associated with poor outcomes. Before the advent of targeted therapy, long-term survival rates were limited to approximately 10-20% with chemotherapy alone. However, since the early 2000s, the introduction of TKIs, including imatinib, second-generation agents such as dasatinib and nilotinib, and the third-generation agent ponatinib, along with immunotherapy such as blinatumomab, has significantly improved survival outcomes in this population [9-11].

Ph+ ALL is an aggressive B-cell malignancy caused by the *BCR-ABL1* tyrosine kinase oncoprotein [9]. Age is the main risk factor [11]. It occurs in 2-5% of children with ALL but in over 50% of adults older than 60, making it the dominant subtype in older patients [9-11]. Overall, about half of cases are diagnosed in patients under the age of 20, so children still represent a large proportion of total cases [9-11]. Approximately 80% of patients have an association with *IKZF1* alterations [12]. Ph+ ALL is more common in Hispanic and Native American populations, partly because of *CRLF2* rearrangements [12]. Inherited variants in genes such as *ARID5B*, *IKZF1*, *CEBPE*, *CDKN2A*/*CDKN2B*, *PIP4K2A*, and *ETV6* increase ALL risk [11]. Males are slightly more affected than females (with a male-to-female ratio of 55:45) [13]. Although no single environmental cause has been confirmed, ionizing radiation, pesticides, and certain occupational exposures are associated with childhood leukemia risk [11,14-17]. The disease arises in bone marrow and may spread to the CNS, testes, liver, spleen, and lymph nodes [2,10]. Diagnosis requires ≥25% lymphoblasts in bone marrow [16].

According to the WHO classification of 2016, leukemia falls under the designation of B-ALL with t(9;22)(q34;q11.2); *BCR-ABL*, which was updated to B-ALL with *BCR::ABL1* fusion in 2022 [10].

Pathogenesis and pathophysiology

B-acute lymphoblastic leukemia (B-ALL) is a clonal malignancy arising from immature B-lineage lymphoid precursor cells; it primarily involves the bone marrow and peripheral blood and most commonly occurs in children and adolescents [2]. The disease is characterized by recurrent numerical and structural chromosomal abnormalities, including aneuploidy and chromosomal rearrangements, which result in oncogene dysregulation. An example is Philadelphia chromosome-positive B-ALL (Ph+ B-ALL), which arises from immature B-lymphoid precursor cells. These leukemic blasts typically express B-cell-associated markers, including CD19 and CD10, as well as terminal deoxynucleotidyl transferase, reflecting their arrested state of early lymphoid differentiation [18].

The Philadelphia chromosome is the most frequent cytogenetic abnormality observed in adult patients with ALL, occurring in approximately 20-30% of adult cases, compared with roughly 5% of pediatric cases [4]. The resulting *BCR-ABL1* fusion protein encodes a constitutively active tyrosine kinase that drives leukemogenesis through persistent activation of downstream signaling pathways involved in cellular proliferation, survival, and resistance to apoptosis while simultaneously inhibiting normal cellular differentiation.

In this patient, FISH analysis confirmed the presence of the *BCR-ABL1* rearrangement, establishing the diagnosis of Ph+ B-ALL. The unchecked tyrosine kinase activity promoted rapid expansion of malignant lymphoblasts within the bone marrow, leading to displacement and suppression of normal hematopoiesis. The severe anemia observed at presentation (hemoglobin: 5 g/dL) likely accounted for the patient’s symptoms of dizziness and fatigue, whereas the marked leukocytosis with a high circulating blast percentage (54%) reflects extensive marrow involvement and peripheral spillover of leukemic cells.

Comparative analysis of our case with the existing literature

Clinical Presentation

Ph+ B-ALL is the most common subtype of ALL, making up about 50% of cases in patients above 50 years old [9]. ALL typically presents with a bimodal distribution, affecting patients around 5 years old and around 50 years old [3,10]. The typical presentation of this cancer involves constitutional symptoms such as fever, night sweats, and weight loss [3,10]. Many patients also exhibit petechiae and very low blood counts [3,10]. These findings are consistent with our patient’s presentation of fatigue, dizziness, shortness of breath, and an initial hemoglobin level of 5 g/dL. However, she did not have hepatosplenomegaly or lymphadenopathy.

Diagnostic Workup

Ph-positive ALL is a hematologic malignancy caused by a translocation of chromosomes 9 and 22, t(9;22)(q34;q11), the most common cytogenetic abnormality in this disease [19]. ALL in children often has a more favorable genetic profile, such as hyperdiploidy, *ETV6::RUNX1* rearrangement/t(12;21), and a lower incidence of *BCR::ABL1* translocation [3,10]. Diagnosis involves flow cytometry, cytogenetic and molecular analysis, such as FISH, and peripheral blood smears [3,10]. One challenge of this leukemia is the common presence of extramedullary disease, especially involving the CNS, emphasizing the importance of proper CNS prophylaxis in treatment plans [3,10]. Studies have shown poor prognosis of Ph+ B-ALL involving gene deletions in *IKZF1* and *CDKN2A/2B*, also emphasizing the need for molecular analysis during diagnostic workup [3,10]. The peripheral smear in our patient showed circulating blasts and immature granulocytes, including large lymphoblasts with a high nuclear-to-cytoplasmic ratio, fine chromatin, and scant cytoplasm, consistent with ALL. Flow cytometry of peripheral blood confirmed B-lymphoblastic leukemia with 54% blasts. Confirmation with FISH testing was also positive for *BCR-ABL1* rearrangement, establishing Ph+ B-ALL. Additionally, FISH analysis demonstrated *IGH* locus disruption. *IGH* rearrangements are well-described in B-cell malignancies and may reflect additional genomic complexity, although their clinical significance in Ph+ B-ALL remains variable.

Management

Before the development of TKIs, the only curative treatment was an allogenic stem cell transplant, and very few patients survived without it [9]. The combination of a TKI and blinatumomab immunotherapy allows for survival rates of approximately 75-80%, reducing the burden of systemic chemotherapy side effects [9]. However, pediatric patients have much higher success rates with treatment than adults, with studies showing over 80% survival in children compared with about 50% in adults [3,10]. These differences stem from factors such as comorbid health conditions that make adults less tolerant of chemotherapy side effects [3,10]. Studies also suggest that the genetic profile of ALL in adults contains features that are less favorable, placing them at higher risk of chemoresistance [3,10]. Most patients undergo a multidrug hyper-CVAD treatment plan in combination with high-dose methotrexate and cytarabine [3,10]. The tumor board panel for our patient ultimately decided that, given her frailty and comorbidities, intensive hyper-CVAD-based induction would be too high-risk. The consensus favored a lower-intensity induction strategy combining mini-CVD with dasatinib because of its CNS penetration and established efficacy in Ph+ B-ALL.

Outcome and Follow-Up

In recent years, this subtype of B-ALL has shown a more promising prognosis, with many patients achieving full remission [9]. With current therapy regimens, Ph-positive ALL has survival rates approaching 90% in some studies [3,10]. Studies have shown that dasatinib combined with hyper-CVAD produced a complete response rate of 96% and a five-year survival rate of 46%, compared with imatinib combined with hyper-CVAD, which had a complete response rate of 93% and a five-year survival rate of 43% [3,10]. With low-intensity therapies such as the one our patient received, similar long-term survival rates of approximately 36% with dasatinib have been reported [3,10]. The patient tolerated induction therapy without major infectious complications. Cytopenias were managed with red blood cell transfusions and growth factors. Atrial fibrillation with rapid ventricular response during hospitalization was managed successfully with rate-control medications. Because of thrombocytopenia, her usual anticoagulation regimen was adjusted with the intention of restarting it once the platelet count normalized. At the second tumor board discussion, our patient achieved hematologic recovery and clearance of morphologic disease on bone marrow biopsy, as well as normalized platelet counts and hemoglobin levels consistent with remission. Cerebrospinal fluid cytology and flow cytometry were negative for malignant involvement. The patient received home health services at discharge, along with scheduled hematology follow-up for close monitoring. The team decided to continue with dasatinib 140 mg daily, maintain antimicrobial prophylaxis with acyclovir and trimethoprim-sulfamethoxazole, and perform serial *BCR-ABL1 *quantitative PCR for measurable residual disease surveillance.

Learnings from this case

This case illustrates several clinically relevant lessons in the management of Ph+ B-ALL in elderly patients with comorbidities. First, it highlights how advanced age and medical complexity should not be interpreted as contraindications to effective leukemia treatment but rather as factors necessitating individualized treatment adaptation. In this patient, the presence of cardiovascular disease, pulmonary hypertension, and chronic thromboembolic disease influenced therapy selection and supported the use of a reduced-intensity regimen instead of hyper-CVAD-based induction.

Second, the combination of low-intensity chemotherapy with TKIs demonstrated the ability to achieve morphologic remission while maintaining acceptable tolerability, reinforcing the evolving role of targeted therapies in frail populations. This approach highlights the feasibility of balancing oncologic efficacy with minimal treatment-related morbidity.

Third, multidisciplinary tumor board evaluation played a critical role in integrating oncologic, cardiovascular, and hematologic considerations to guide patient-centered decision-making. Such collaboration is particularly valuable in complex hematologic malignancies where competing risks influence treatment strategy.

Finally, this case highlighted the importance of continued adherence to core ALL management principles, including CNS prophylaxis and measurable residual disease surveillance, even with de-intensified treatment frameworks. Collectively, these observations support the expanding role of personalized and comorbidity-adapted therapeutic approaches in improving outcomes for elderly patients with Ph+ B-ALL.

## Conclusions

Ph+ B-ALL is considered a high-risk hematologic malignancy, especially in elderly patients with comorbidities who are not candidates for intense cytotoxic medications or allogeneic stem cell transplantation. This case highlights the effectiveness of reduced-intensity chemotherapy in combination with a TKI for disease control in frail patients. Our patient achieved morphologic remission following induction with mini-CVD and dasatinib despite advanced age and cardiovascular comorbidities, emphasizing the importance of individualized risk-based treatment plans. The positive association between the incidence of Ph+ ALL and advanced age accentuates the need to develop more efficacious therapeutic approaches for this population. This case supports the growing research on TKI-based treatment regimens in the management of elderly patients with Ph+ B-ALL and reinforces the critical priority for continued study in personalized therapy that balances effectiveness and safety in high-risk populations.
